# Iron overload induced submandibular glands toxicity in gamma irradiated rats with possible mitigation by hesperidin and rutin

**DOI:** 10.1186/s40360-024-00744-8

**Published:** 2024-02-27

**Authors:** Salwa Farid Ahmed, Eman M. F. El-Maghraby, Maha M. Rashad, Dina W. Bashir

**Affiliations:** 1 Health Radiation Research Department, National Centre for Radiation Research and Technology, Egyptian Atomic Energy Authority, Cairo, Egypt; 2 Biochemistry and Molecular Biology Department, Faculty of Veterinary Medicine, Cairo University, Giza, Egypt; 3 Cytology and Histology Department, Faculty of Veterinary Medicine, Cairo University, Giza, Egypt

**Keywords:** Ionizing radiation, Iron overload, Rutin, Hesperidin, SOD, BRCA1, TNF-α, AKT, NF-κB

## Abstract

**Background:**

Radiation triggers salivary gland damage and excess iron accumulates in tissues induces cell injury. Flavonoids are found in some fruits and are utilized as potent antioxidants and radioprotective agents. This study aimed to evaluate the antioxidant and anti-inflammatory effects of hesperidin and rutin on gamma radiation and iron overload induced submandibular gland (SMG) damage and to evaluate their possible impact on mitigating the alteration in mTOR signaling pathway and angiogenesis.

**Methods:**

Forty-eight adult male Wistar albino rats were randomly assigned to six groups: group C received a standard diet and distilled water; group H received hesperidin at a dose of 100 mg/kg; four times a week for four weeks; group U received rutin at a dose of 50 mg/kg; three times a week for three weeks; group RF received a single dose (5 Gy) of gamma radiation followed by iron at a dose of 100 mg/kg; five times a week for four weeks; group RFH received radiation and iron as group RF and hesperidin as group H; group RFU received radiation and iron as group RF and rutin as group U. SMG specimens from all groups were removed at the end of the experiment; and some were used for biochemical analysis, while others were fixed for histological and immunohistochemical examination.

**Results:**

In the RF group, several genes related to antioxidants (Nrf-2 and SOD) and DNA damage (BRCA1) were significantly downregulated, while several genes related to inflammation and angiogenesis (TNFα, IL-1β and VEGF) and the mTOR signaling pathway (PIK3ca, AKT and mTOR) were significantly upregulated. Acinar cytoplasmic vacuolation, nuclear pyknosis, and interacinar hemorrhage with distinct interacinar spaces were observed as histopathological changes in SMGs. The duct system suffered significant damage, eventually degenerating entirely as the cells were shed into the lumina. VEGF and NF-κB were also significantly overexpressed. Hesperidin and rutin cotreatment generated partial recovery as indicated by significant upregulation of Nrf-2, SOD and BRCA1 and considerable downregulation of TNF-α, IL-1β, VEGF, PIK3ca, AKT, and mTOR. Although some acini and ducts continued to deteriorate, most of them had a normal appearance. There was a notable decrease in the expression of VEGF and NF-κB.

**Conclusions:**

In γ-irradiated rats with iron overload, the administration of hesperidin and rutin may mitigate salivary gland damage.

## Introduction

For patients with head and neck cancer, radiation therapy is typically utilized as the first line of treatment, either curative or palliative. It is frequently used in combination with immunotherapy, chemotherapy, and surgery [1]. Radiation-induced injury is mainly attributed to reactive oxygen species (ROS), which can damage DNA followed by a cascade of biological changes that can ultimately lead to cell death. The extent to which cells are susceptible or resistant to radiation injury depends on the degree of differentiation, rate of division and cumulative and fractional radiation doses [2]. Among the toxicities resulting from head and neck radiation, dry mouth is diagnosed in most treated patients and has a significant negative impact on quality of life. The size of the salivary glands at which patients receive threshold doses determines the level of recovery that can reach full recovery [3]. Mouthwashes, gums or sweets containing xylitol, and saliva substitutes are some of the treatments used to treat dry mouth; nevertheless, their effectiveness is limited [4]. It could be better when dealing with postradiation complications in salivary glands to develop new strategies based on regeneration of the salivary gland itself rather than the use of substitutes for its products.

Another element that may cause damage to the salivary glands is iron. Iron is the primary component of reduction-oxidation reactions that provide energy and is crucial to many other fundamental activities; thus, iron is an element that is necessary for life. It is involved in the process of oxygen transport and release of hemoglobin and myoglobin, respectively and is required for DNA synthesis [5]. Since humans do not have a physiological system for eliminating excess iron, excess iron can be quite harmful. When the transferrin binding capacity is surpassed, iron overload occurs. Acute iron overload was caused by taking too many iron-containing supplements, but chronic iron overload resulted from prolonged intake of iron-containing supplements. When non-transferrin bound iron accumulates as free iron in tissues, it can be toxic and lead to cellular damage and organ failure [6].

Anemia linked to cancer is a symptom that can follow the development of cancer and is more frequently identified in individuals in later stages of the disease. According to Madedu et al. [7], anemia is mostly caused by the chronic inflammatory state of advanced cancer patients. Therapeutic approaches for such anemia should focus on the various factors that cause the disease, such as iron or blood transfusions, nutritional supplements, anti-inflammatory medications, and erythropoietic agents, since neoplastic illness is frequently incurable [8]. The most recent National Comprehensive Cancer Network NCCN guidelines [9] state that RBC transfusions should be considered in patients with symptomatic anemia, high-risk patients (such as those receiving high-dose chemotherapy or radiation with a cumulative decrease in Hb levels), or asymptomatic patients with comorbidities (such as heart disease or cerebral vascular disease) rather than those based on a specific threshold value of Hb.

The early signs of iron overload are typically nonspecific and include abdominal upset and exhaustion. In certain situations, there are no symptoms at all, which makes it difficult to detect the condition early on until serious organ damage becomes clinically apparent and increases mortality [10]. To increase survival, efficient iron chelators must be developed for patients with transfusion-dependent chronic anemia because they have a greater risk of iron overload. Flavonoids, as powerful antioxidants, protect tissues against oxidative stress induced by free radicals through direct scavenging of reactive oxygen and nitrogen species in addition to stimulating antioxidant enzymes [11].

Hesperidin, a characteristic bioflavonoid of citrus fruits, is found in abundant quantities in orange, grapefruit, tangerine, lime, and lemon [12]. It has many biological and pharmacological properties including antioxidant [13], anti-inflammatory [12], anticancer [14], and radioprotective [15] effects.

Rutin, another bioflavonoid, is a combination of the flavonol quercetin and disaccharide rutinose abundantly found in plants such as buckwheat, apricots, cherries, grapes, grapefruit, onion, plums, oranges, and apple in addition to plant-based drinks such as green tea [16]. Rutin has been shown to have numerous biological effects, including antioxidant [17], anti-inflammatory [18], anticancer [19], and radioprotective [20] activities.

Despite the aforementioned findings, the radioprotective effect of flavonoids on irradiated salivary glands with iron overload has not yet been studied. Therefore, the present study aimed to evaluate the antioxidant and anti-inflammatory effects of hesperidin and rutin on gamma radiation and iron overload induced SMGs damage and to evaluate their possible impact on mitigating the alteration in mTOR signaling pathway and angiogenesis.

## Methods

### Chemicals

Iron; CosmoFer 50 mg/ml (Iron III-hydroxide dextran complex) solution for infusion and injection (Pharmacosmos UK Limited) was purchased from the Egyptian Pharmacists Company, Egypt. Hesperidin was purchased from Sigma -Aldrich (USA) and was dissolved in distilled water. Rutin was purchased from Sigma -Aldrich (USA) and was dissolved in distilled water.

### Irradiation

In the course of the study, the rats were subjected to a single dose of 5 Gy of whole body gamma radiation at the National Centre for Radiation Research and Technology, Egyptian Atomic Energy Authority, Cairo, Egypt (dose rate 0.36 Gy/min) using ^137^Cesium gamma cell-40.

### Ethical consideration and animal grouping

Forty-eight mature male Wistar albino rats weighing 180–200 g were chosen for the experiment. The animals were obtained from the animal house of the National Centre for Radiation Research and Technology. The animals were maintained in typical laboratory settings with free access to water, a standard diet, and a 12-hour light/dark cycle. The temperature was kept at 25 ± 3 °C and the humidity was kept at 55% ± 5%. The rats were allowed to acclimate for ten days before the trial began. With protocol number Vet CU 08/07/2023/710, the experiment was carried out in accordance with the guidelines approved by Cairo University’s Faculty of Veterinary Medicine’s Institutional Animal Care and Use Committee.

The rats were randomly assigned to six groups (*n* = 8 in each group). The control group (C); served as an untreated negative control and were fed a standard diet and distilled water. Rats in the hesperidin group (H) were given 100 mg/kg hesperidin intraperitoneally four times a week for four weeks [10]. The rats in the rutin group (U) were orallyadministered 50 mg/kg rutin three times a week for three weeks [21]. Rats in the radiation + iron group (RF) received intraperitoneal iron injection five times a week for four weeks after receiving a single 5 Gy dose of gamma radiation [22]. Rats in the radiation + iron + hesperidin group (RFH) were received radiation and iron as the RF group and hesperidin as the H group. Rats in the radiation + iron + rutin group (RFU) were received radiation and iron as the RF group and rutin as the U group.

### Animal euthanasia

To obtain the tissue specimens at the end of the trial, the rats were euthanized by intraperitoneal injection of the anesthetic thiopental sodium (EIPICO, Egypt) at a dose of 50 mg/kg followed by cervical dislocation for death confirmation. After carefully removing the SMG specimens from each group, one gland from each rat was placed in a plastic bag and kept at -80 °C for biochemical analysis. The remaining glands were fixed in 10% neutral buffer formalin for 48 h. The selected samples were subsequently washed, dehydrated in ascending grades of ethyl alcohol, cleared in xylene, and embedded in paraffin wax for histology and immunohistochemistry.

### Biochemical evaluation (real-time PCR analysis)

Using GAPDH as a housekeeping gene, quantitative real-time PCR (qRT-PCR) was used to evaluate the relative salivary mRNA expression levels of the Nrf-2, SOD, BRCA, TNF-α, IL-1β, VEGF, PIk3ca, AKT, and mTOR genes [23]. A total RNA extraction kit (Vivantis, Malaysia) was used to extract total RNA from approximately 50 mg of salivary gland tissue. The content and purity of the RNA were verified using NanoDrop technology [24]. M-MuLV reverse transcriptase (NEB#M0253) was used for RT-PCR. SYBR green PCR Master Mix (Thermo Scientific, Cat. No. K 0221) was used for the RT-PCR analysis [25]. Table 1 lists the sequences of primers used. Primer3 (v. 0.4.0) software, available for free online at http://bioinfo.ut.ee/primer3-0.4.0, was used to construct primer sets. Three biological replicates were used for each qPCR, and each biological replication was subjected to three assessments [26]. Template-free negative controls were used [27]. The relative transcription levels were calculated using the comparative 2 − ΔΔCT method [28].

**Table 1:** Sequence of Primers used for real-time PCR

Genes	Gene description	Accession number	Primer Sequence
Nrf 2	Nuclear factor, erythroid 2-like 2	NC_005102.4	F: -5′-GGCCCTCAATAGTGCTCAG‐3′ R: -5′-TAGGCACCTGTGGCAGATTC‐3′
SOD	Super oxide dismutase	NM_017050	F: 5′- -GCAGAAGGCAAGCGGTGAAC‐3′ R:5′‐ TAGCAGGACAGCAGATGAGT-3′
BRCA	Breast cancer gene 1	AF036760.1	F: 5′- CCCCTGATCCCGATAATGCT‐3′ R: 5′- TGTGAAGGGCTGCTCTTGTA ‐3′
TNF-α	Tumor necrosis factor alpha	NM_012675.3	F: 5′-ACACACGAGACGCTGAAGTA-3′ R: 5′-GGAACAGTCTGGGAAGCTCT-3′
IL-1β	Interleukin-1 beta	NM_031512.2	F: 5′- TTGAGTCTGCACAGTTCCCC ‐3′ R: 5′- GTCCTGGGGAAGGCATTAGG ‐3′
VEGF	Vascular endothelial growth factor A	NM_001287110.1	F: 5′- TTCCTGTAGACACACCCACC ‐3′ R: 5′- TCCTCCCAACTCAAGTCCAC ‐3′
AKT	AKT Serine/Threonine Kinase 1	NM_033230.3	F: 5′- CTGCCCTTCTACAACCAGGA‐3′ R: 5′- GTGCTGCATGATCTCCTTGG ‐3′
mTOR	Mechanistic target of Rapamycin Kinase	NM_019906.2	F: 5′- TCTGCACTTGTTGTTGCCTC‐3′ R: 5′- ACAATCGGGTGAATGATGCG ‐3′
PIk3ca	Phosphatidylinositol-4,5-bisphosphate 3-kinase, catalytic subunit alpha	NM_133399.3	F: 5′- TAGTGTCCGGGAAAATGGCT ‐3′ R: 5′- GGCATGCTCTTCGATCACAG ‐3′
GAPDH	Glyceraldehyde3-phosphate dehydrogenase	NC_005103.4	F: − 5′-ACCACAGTCCATGCCATCAC-3′ R: − 5′-TCCACCACCCTGTTGCTGTA-3′

### Histopathological examination

Hematoxylin and eosin (H&E) staining was applied to sections measuring 3–4 μm after deparaffinization for examination under a light microscope [29].

For vascular endothelial growth factor (VEGF) immunostaining, the sections were deparaffinized with xylene and then hydrated in a series of descending grades of alcohol. The sections were then briefly rinsed with tap water and phosphate buffered saline (PBS), at a pH of 7.4. The sections were incubated in methanol supplemented with 0.3% hydrogen peroxide (H_2_ O_2_) for 10 minutes. Normal serum was used for 10 minutes to prevent nonspecific protein binding. The sections were treated with polyclonal (anti vascular endothelial growth factor, Santa Cruz Biotechnology, Santa Cruz, CA, USA) primary antibodies for 12 hours at 4°C. After three 5-minute rinses with PBS, the sections were incubated with biotinylated secondary antibodies for 10 minutes and with a streptavidin-enzyme conjugate for 5 minutes. Following peroxidase activation with 3, 3’-diaminobenzidine for 10 min, the antibody-localized antigen was observed to cause a brownish discoloration. Finally, the sections were counterstained lightly with hematoxylin [30].

For nuclear factor-kappa B (NF-κB-p65) the manufacturer’s protocol (catalogue no. E-AB-32,232; Elabscience Biotechnology Inc., USA) for immunohistochemistry was followed. Dewax and hydrate the portions briefly. For 10 min, the sections were incubated with E-IR-R217C (3% H_2_O_2_) to remove endogenous peroxidase activity. E-IR-R217A (usual goat blocking buffer) was added, after which the sections were incubated for 30 min at 37 °C. The primary polyclonal anti-NF-B-p65 antibody (from a mouse or a rabbit) was added at a dilution of 1:100, and the samples were incubated for 1.5 h at 20–37 °C or overnight at 4 °C (before warming at 37 °C for 30 min). The area was dried with absorbent paper. E-IR-R217B (polyperoxidase-anti-mouse/rabbit IgG) was added, and the samples were incubated for 20 min at 37 °C or room temperature. The sections were washed in PBS for two minutes following each step. For every 1 mL of E-IR-R217E (DAB Substrate), 1 drop of E-IR-R217D (DAB concentration) was added, and the mixture was combined well. After the DAB staining phase was controlled; a tan or brownish yellow colour was observed. After counterstaining, dewatering, and sealing, the pieces were washed in deionized water to stop the chromogenic process.

Immunohistochemically stained sections were evaluated using a Leica Qwin 500 analyser computer system (Leica Microsystems, Switzerland). The area percentage of immunohistochemical staining in five fields from different slides in each group at a magnification of X400 was measured using light microscopy, which was subsequently transmitted to the monitor’s screen. For each specimen, the mean value and standard error (SE) were calculated and statistically analyzed.

### Statistical analysis

The means ± SE were used to express the results. One-way analysis of variance was used to determine the significance of the difference in the means between the groups, followed by a post-hoc test for the least significant difference using SPSS version 25. A difference was considered to be statistically significant if the *p*-value was less than 0.05.

## Results

### Biochemical results

#### The effect of hesperidin and rutin on several antioxidant- and DNA damage-related genes

**Fig. 1:**
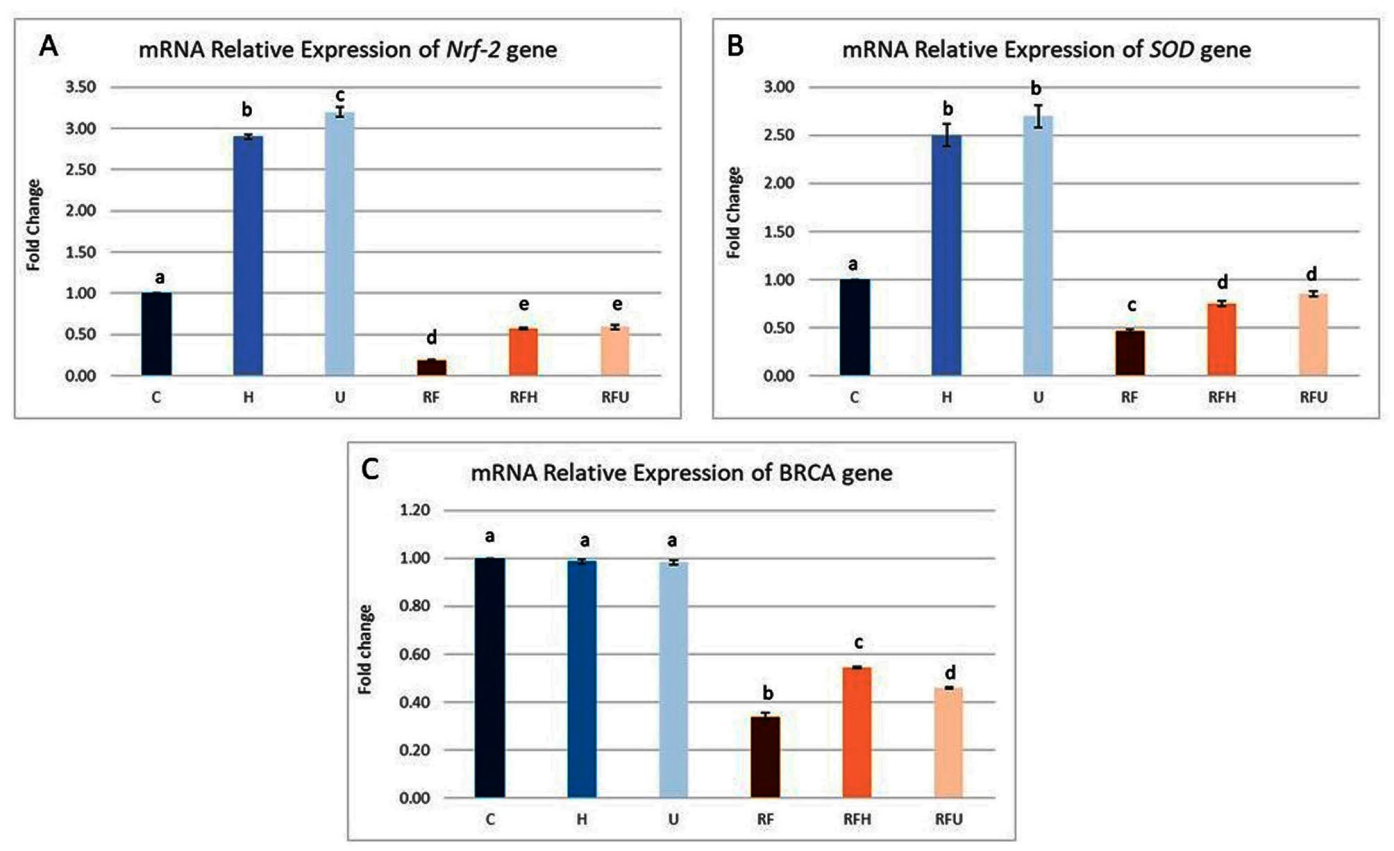
The effects of hesperidin, and rutin on mRNA expression of several antioxidant- and DNA damage- related genes. (**A**) Nrf-2, (**B**) SOD and (**C**) BRCA1 expression in submandibular gland of gamma irradiated albino rats with iron overload. Means with different superscripts (^a, b, c, d^) differ significantly at *p* < 0.05

Figure 1 illustrates how rutin and hesperidin affect the mRNA expression of the Nrf-2, SOD and BRCA1 genes in SMGs exposed to gamma radiation and iron overload. The RF group exhibited significant decrease in the relative expression of Nrf-2, SOD, and BRCA1 to 0.19, 0.46, and 0.34-fold respectively compared with those in the negative control group. On the other hand, cotreatment with hesperidin significantly induced the upregulation of Nrf-2, SOD, and BRCA1 expression compared with that in the RF group. Additionally, rutin cotreatment significantly improved the expression of these genes as shown in Fig. 1. The groups treated with hesperidin (RFH) or rutin (RFU) showed a discernible rise in Nrf-2, SOD, and BRCA1 expression; nevertheless, these levels did not reach normal.

#### The effect of hesperidin and rutin on several inflammation- and angiogenesis-related genes

**Fig. 2:**
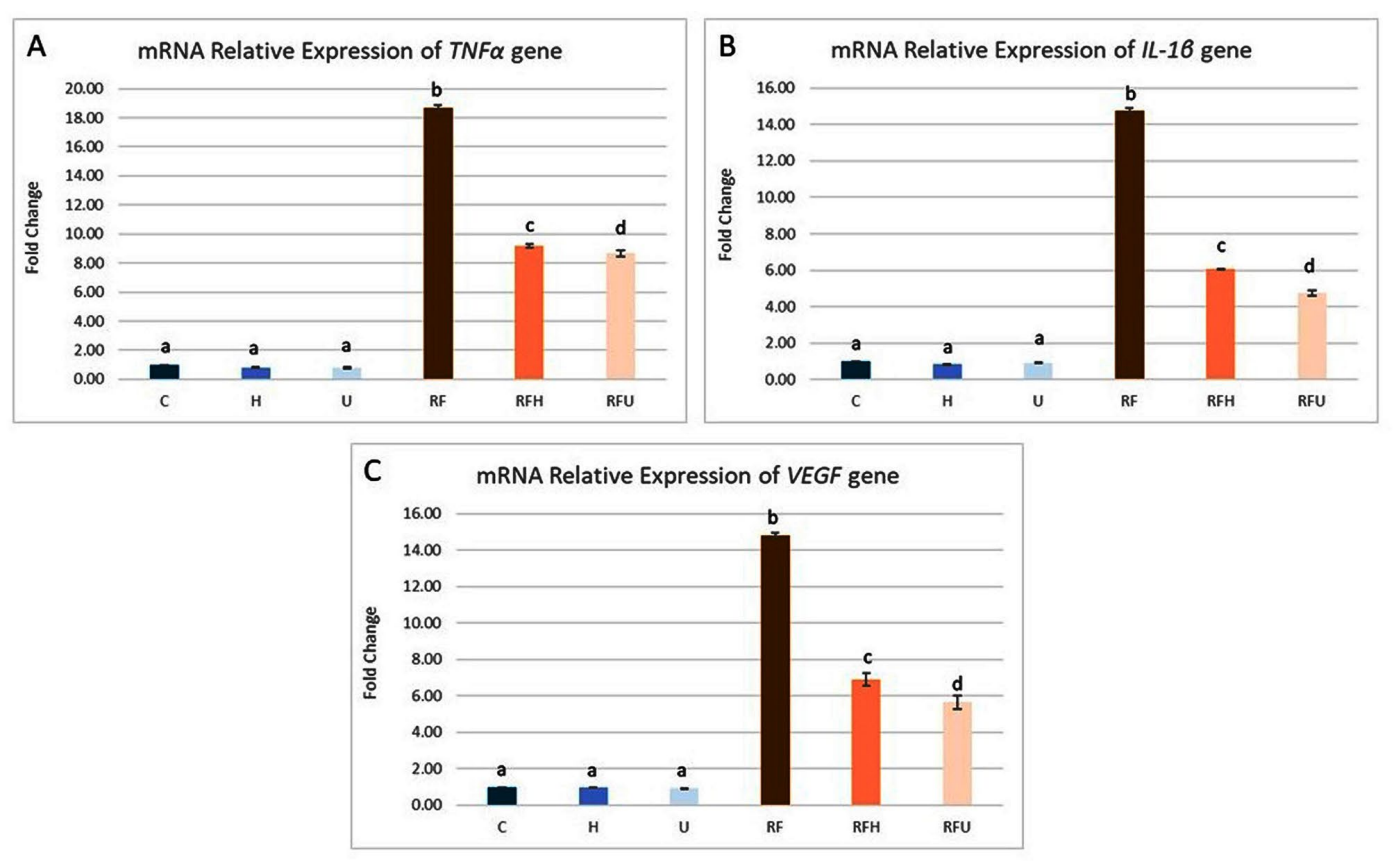
The effects of hesperidin and rutin on the mRNA expression of several inflammation- and angiogenesis-related genes (**A**) TNFα, (**B**) IL-1β and (**C**) VEGF expression in submandibular gland of gamma irradiated albino rats with iron overload. Means with different superscripts (^a, b, c, d^) differ significantly at *p* < 0.05

The impact of rutin and hesperidin on the mRNA expression of TNFα, IL-1β, and VEGF genes in SMGs subjected to gamma radiation and iron overload is demonstrated in Fig. 2. TNFα, IL-1β, and VEGF gene expression was significantly upregulated in the RF group to 18.67, 14.76, and 14.80-fold, respectively, when compared with the control group. Compared with those in the RF group, the expression of these genes significantly decreased in the RFH and RHU groups. The TNFα, IL-1β, and VEGF gene expression in the RFH and RFU groups was significantly higher than in the control group, as shown in Fig. 2.

#### The effect of hesperidin and rutin on mTOR signaling pathway-related genes

**Fig. 3:**
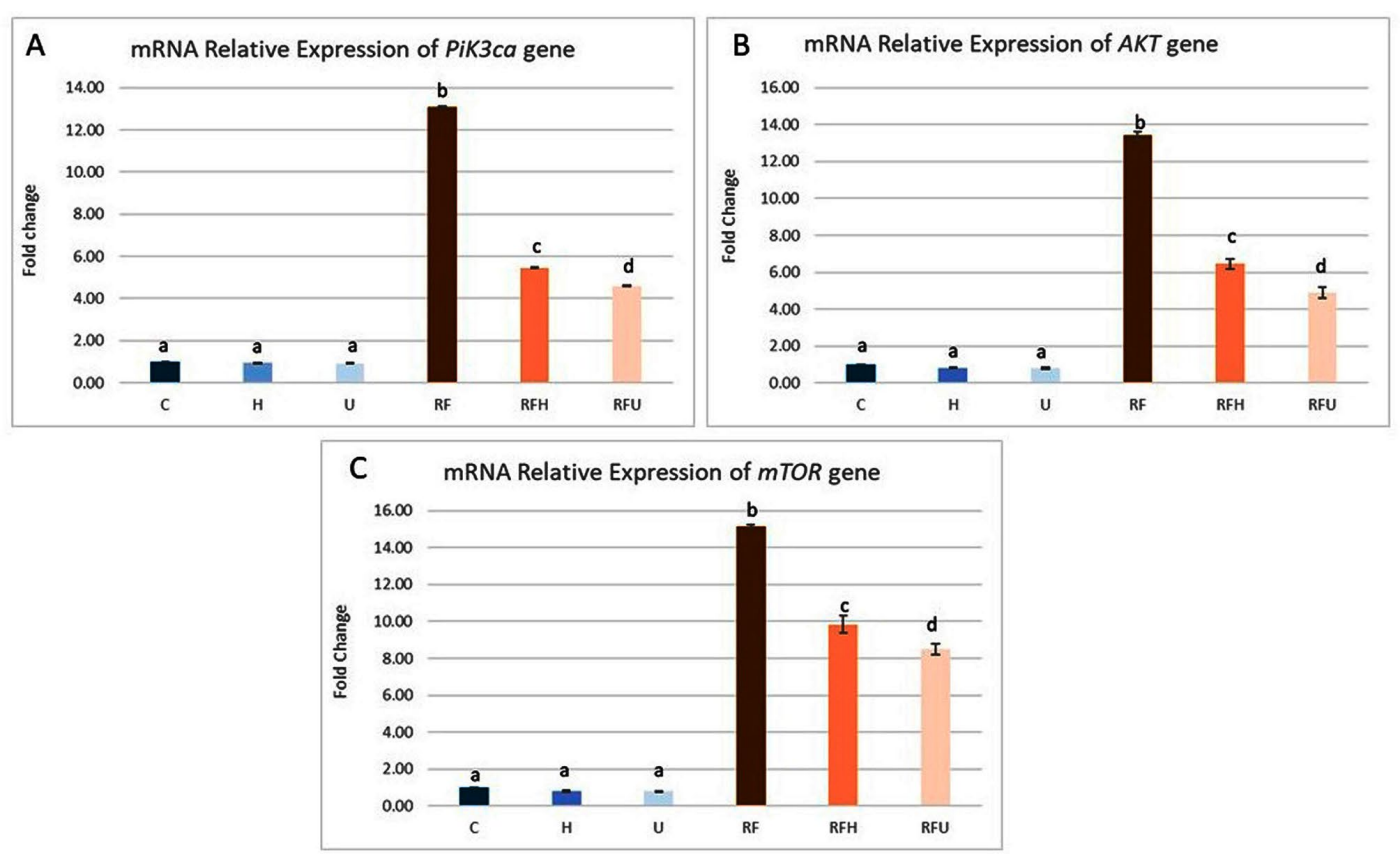
The effects of hesperidin and rutin on the mRNA expression of the PIK3ca, AKT, and mTOR signaling pathway-related genes (**A**) PIk3ca, (**B**) AKT and (**C**) mTOR expression in submandibular gland of gamma irradiated albino rats with iron overload. Means with different superscripts (^a, b, c, d^) differ significantly at *p* < 0.05

In SMGs exposed to gamma radiation and iron overload, Fig. 3 shows the effects of rutin and hesperidin on the mRNA expression of the PIK3ca, AKT, and mTOR genes. The relative gene expression of PIK3ca, AKT, and mTOR was significantly upregulated in the RF group to 15.15, 13.41, and 13.07-fold respectively compared with the control group. Compared with those in the RF group, the expression of these genes significantly decreased in the groups co treated with hesperidin (RFH group) or rutin (RFU group). Although the expression of PIK3ca, AKT and mTOR genes decreased, it was still higher than the control group.

### Histopathological results

#### The effects of hesperidin and rutin on the histological structure

We performed a histological analysis to demonstrate the changes induced by iron overload and gamma radiation on albino rat SMGs, as well as the improvement brought about by hesperidin and rutin cotreatment. The normal histological architecture of the SMGs was shown by examination of H&E- stained sections from the albino rats in the C group, H group, and U group as the secretory unit “acini” appeared to be a mixture of mucous and serous cells.

**Fig. 4:**
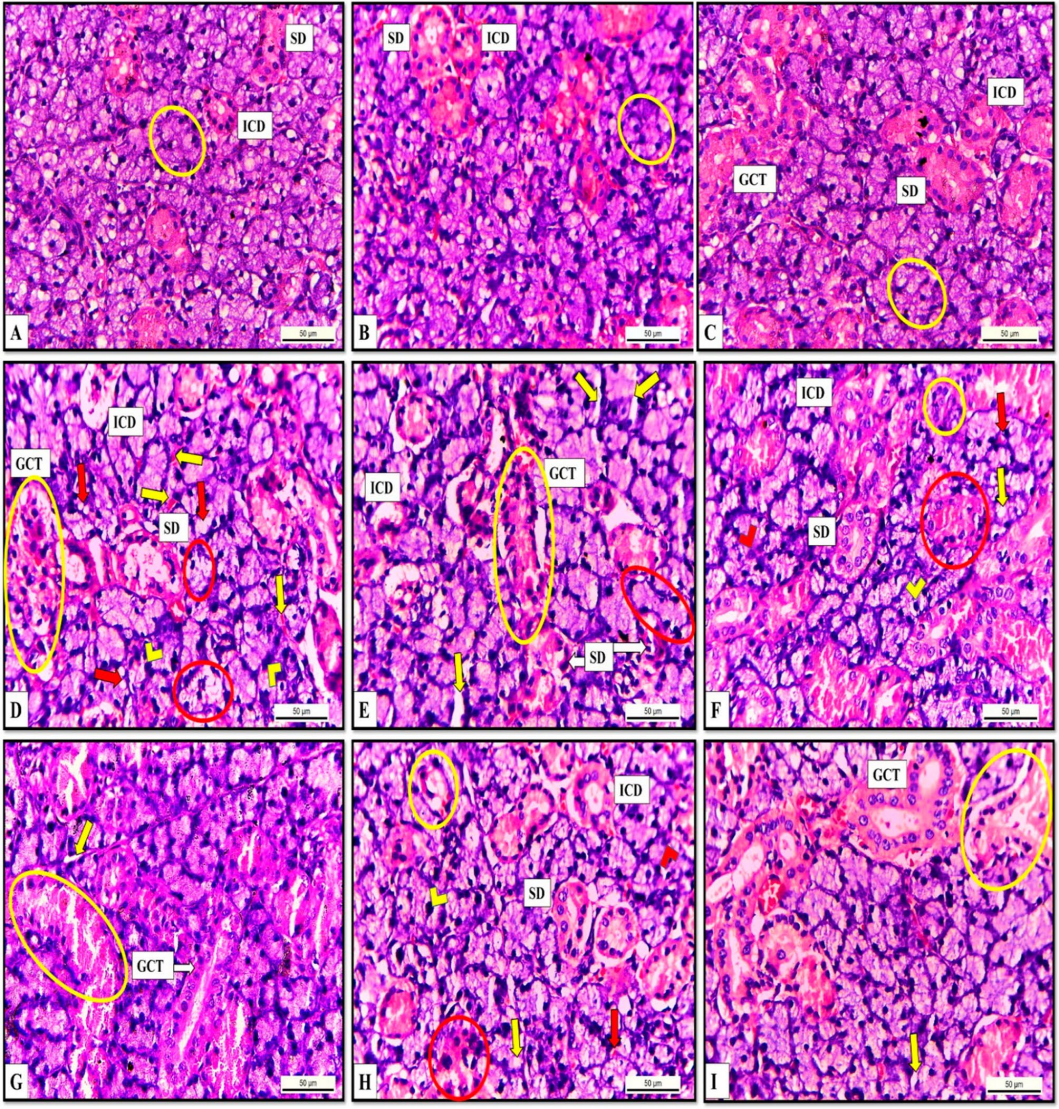
H&E- stained Submandibular gland sections of albino rats (X400). (**A**) group C (**B**) group H (**C**) group U showing normal acinar architecture (circle), intact intercalated duct (ICD), striated duct (SD), and granular convoluted tubule (GCT) (**D-E**) group RF showing (**D**) Acinar cells with cytoplasmic vacuolation (red arrows) and nuclear pyknosis (chevrons), completely degenerated acini with shedding of the nuclei into the lumina (red circles), interacinar hemorrhage (yellow arrows), completely degenerated (ICD) with shedding of their cells into the lumina, (SD) lost the normal arrangement of the ductal cells with some nuclei appeared flattened and with polymorphism, and degenerated (GCT) with sloughing of their epithelial lining into the lumina. (**E**) Several acini appeared without demarcation between their cells (red circle), interacinar spaces (yellow arrows), completely degenerated (ICD) with shedding of their cells into the lumina; lost the normal arrangement of the ductal cells in (SD) and (GCT) with sloughing of their epithelial lining into the lumina leaving empty spaces around them with some nuclei appearing pyknotic, flattened and polymorphic. (**F-G**) group RFH and (**H-I**) group RFU showing partial preservation of the normal architecture (**F-H**) Acinar cells with small areas of acinar cell vacuolation (yellow arrow), some nuclei appeared to be normal vesicular (red chevron) others still pyknotic (yellow chevron), a decreased incidence of interacinar hemorrhage (red arrow), some (ICD), (SD) appeared with nearly normal architecture, some intact epithelial cells with vesicular nuclei while others ICD (yellow circle) SD (red circle) still degenerated with cellular disruption. (**G-I**) Few Interacinar spaces (yellow arrow) and some GCTs returned nearly normal while others (yellow circle) still degenerated

The duct system was normal and intact; the intercalated ducts (ICD) were lined by simple cuboidal cells, the striated ducts (SD) had columnar epithelial cells with rounded to oval nuclei, and the granular convoluted tubules (GCT) contained larger columnar cells (Fig. 4A, B, and C).

In contrast, the SMGs in the RF group exhibited a loss of normal gland architecture characterized by cytoplasmic vacuolation of the acinar cells, nuclear pyknosis and interacinar hemorrhage. While some acini (Fig. 4D) displayed severe degeneration with nuclei shedding into the lumina, others (Fig. 4E) lacked a cell boundary with clear interacinar spaces.

The duct system was severely affected where the ICD completely degenerated with the shedding of cells into the lumina, and the SD and GCT showed loss of the normal arrangement of the ductal cells with sloughing of their epithelial lining into the lumina leaving empty spaces around them. Moreover, some nuclei of the ductal cells appeared pyknotic and flattened and had polymorphisms (Fig. 4D, E).

Examination of the SMGs from the RFH and RFU groups revealed partial preservation of the gland morphology compared with that in the RF group where acinar cell vacuolation, nuclear pyknosis, interacinar spaces and hemorrhage decreased to a great extent. Furthermore, the duct system preserved the normal cellular arrangement, in which some ICDs, SDs, and GCTs appeared nearly normal with intact outlines and epithelial linings with vesicular nuclei while others still degenerated with cellular disruption (Fig. 4F, G, H, I).

#### The effects of hesperidin and rutin on VEGF expression

**Fig. 5:**
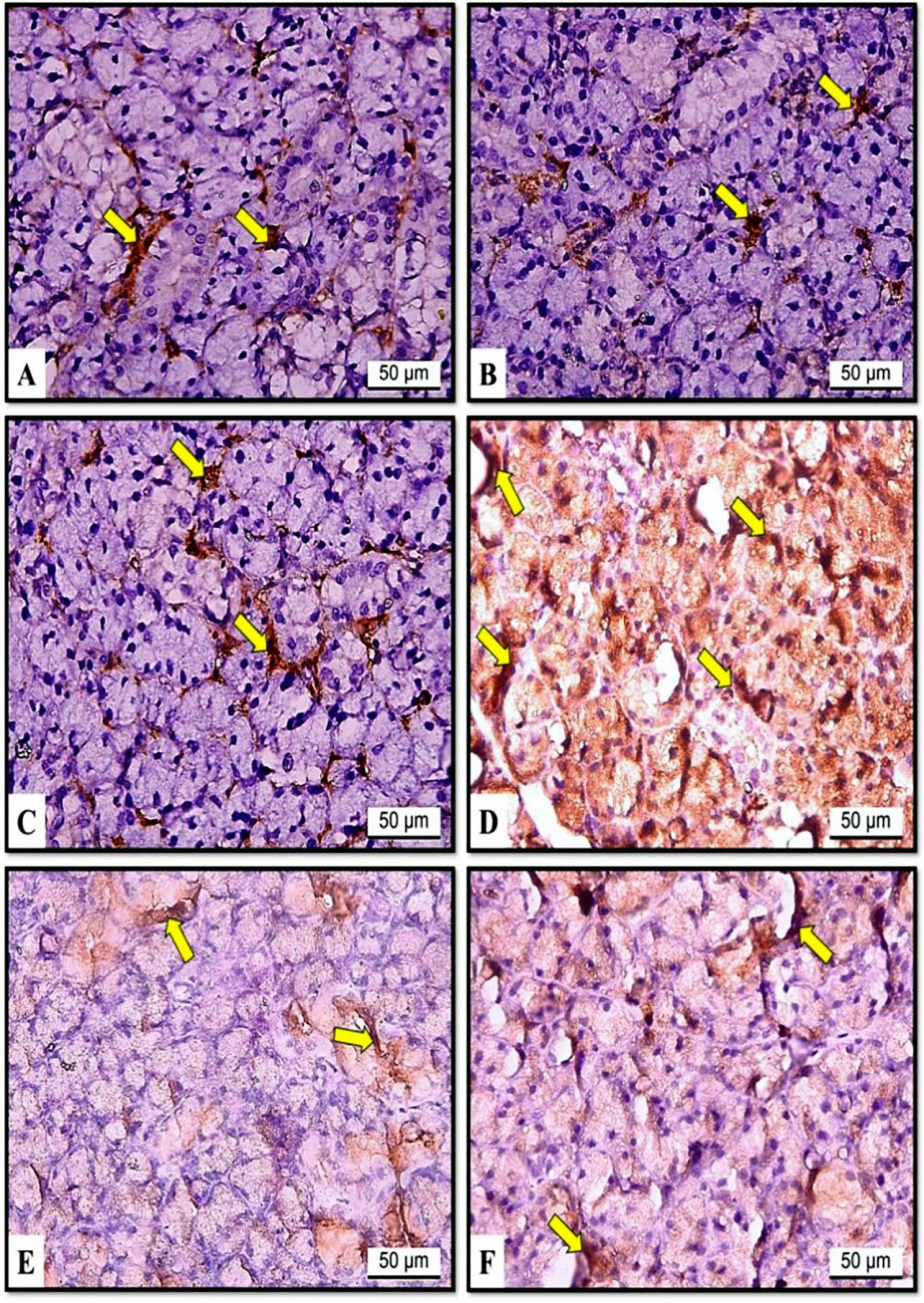
Effects of hesperidin and rutin on VEGF expression in submandibular gland sections of gamma irradiated albino rats with iron overload (X400). (**A**) Group C (**B**) Group H (**C**) Group U showing mild immunoreactivity (arrows) (**D**) Group RF showing intense reaction (arrows) (**E**) Group RFH (**F**) Group RFU showing moderate expression (arrows)

We used immunohistochemistry analysis for VEGF expression to investigate the impact of hesperidin and rutin on the angiogenesis of SMGs in irradiated rats with iron overload. Examination of SMG immunohistochemical sections from the albino rats in the C, H and U groups showed mild expression of VEGF (Fig. 5A, B and C). Group RF demonstrated intense VEGF expression (Fig. 5D). However, moderate expression was seen in both RFH and RFU (Fig. 5E and F).

#### The effects of hesperidin and rutin on NF-κB-p65 expression

**Fig. 6:**
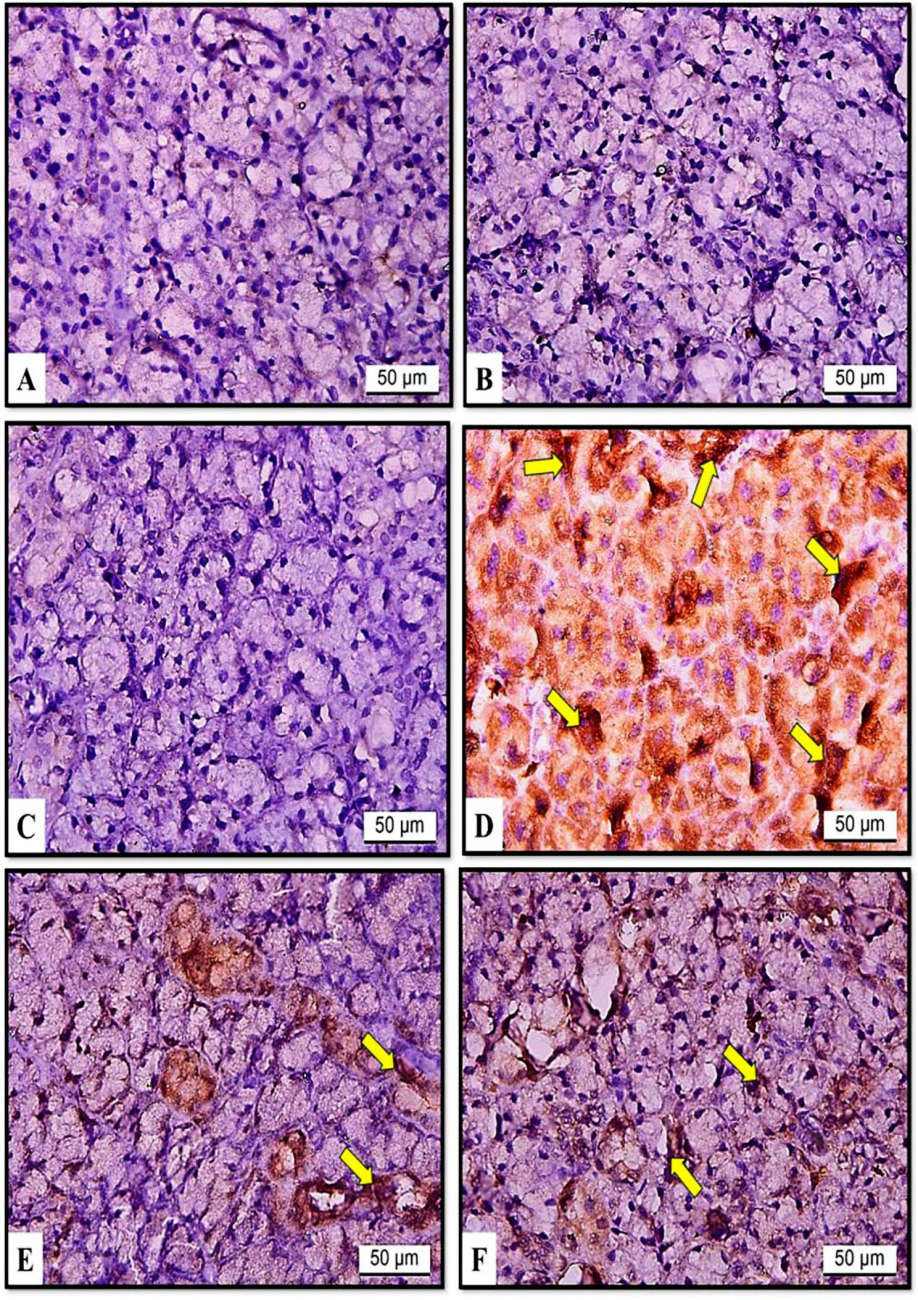
Effects of hesperidin and rutin on NF-κB-p65 expression in submandibular gland sections of gamma irradiated albino rats with iron overload (X400). (**A**) Group C (**B**) Group H (**C**) Group U showing negative expression (**D**) Group RF showing intense immunoreactivity (arrows) (**E**) Group RFH (**F**) Group RFU showing moderate reaction (arrows)

The study employed NF-κB-p65 immunohistochemistry analysis to examine the radioprotective effects of rutin and hesperidin against gamma radiation- and iron overload-induced inflammation in SMGs of rats. The results indicated that NF-κB-p65 was not expressed in the C, H, or U groups (Fig. 6A, B and C). In the RF group, NF-κB-p65 expression was intense (Fig. 6D) in contrast to RFH and RFU groups where moderate expression was identified (Fig. 6E, F).

**Fig. 7:**
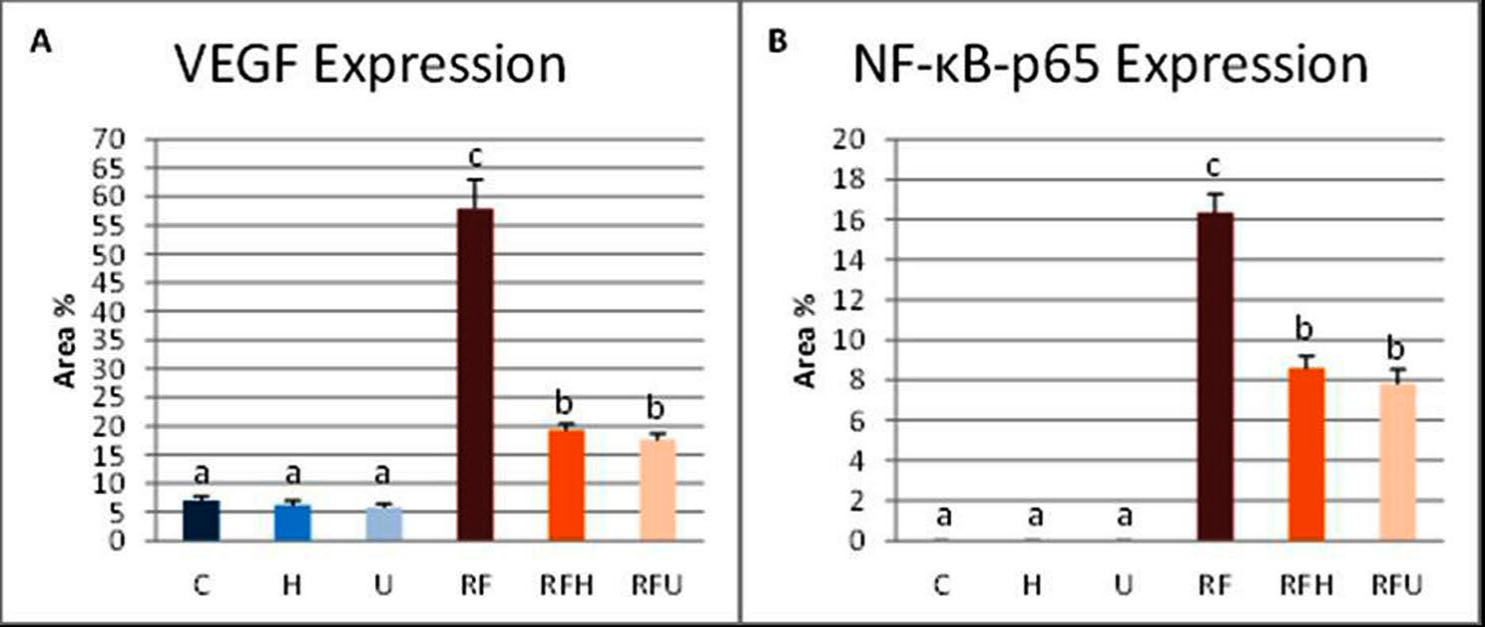
The chart illustrates the quantification (area %) of VEGF and NF-κB-p65 expression. Means with different superscripts (^a, b, c^) differ significantly at *p* < 0.05

The quantification of VEGF and NF-κB-p65 expressions was presented in Fig. 7. Compared with those in the control group, the RF group had significantly greater VEGF expression. In the RFH and RFU groups, VEGF expression was significantly lower than those in the RF group but still significantly higher than those in the control group. Moreover, the data indicated a significant increase in NF-κB-p65 immunoreactivity between the RF group and the control group. Furthermore, the expression of NF-κB-p65 in the RFH and RFU groups was significantly lower than that of the RF group, while it was significantly greater than that of the control group (Fig. 7).

## Discussion

Since the salivary glands are often located within the radiation zone during the treatment of head and neck cancers, which can increase susceptibility to damage and damage resulting from iron overload as a result of frequent blood transfusions in some cases, the development of protective agents against radiation injury and associated comorbidities is necessary.

The present study aimed to evaluate the possible protective effects of some flavonoids (hesperidin, and rutin) on gamma radiation- and iron overload- induced salivary gland damage. The deleterious effect of radiation is attributed to disturbances in the cellular redox state, DNA damage, inflammatory changes, microvascular injury, and impairment of mTOR signaling [31]. Therefore, the relative mRNA expression levels of several antioxidant and DNA damage response genes (Nrf-2, SOD, and BRCA1), inflammation and angiogenesis related genes (TNFα, IL-1β, and VEGF genes), and mTOR signaling related genes (PIK3ca, AKT, mTOR) was evaluated. The present study revealed a significant downregulation of antioxidant-related genes (Nrf-2 and SOD) in response to gamma radiation exposure and iron overload. The depletion of antioxidant enzymes might be due to their consumption by ROS [32]. Such depletion in the antioxidant state is associated with the induction of oxidative stress. According to Karerr et al. [33], 10 days after radiation exposure, MDA, total oxidant status (TOS), and oxidative stress index (OSI) greatly increased while enzyme activities of GPx and CAT decreased. These findings suggest a close relationship between oxidative stress and radiation induced-tissue damage. As reported by Ozgur et al. [34], radiotherapy causes an elevation in salivary and blood gland OSI in rats. Radwan and Karam [35] observed that oxidative stress is linked to radiation induced-intestinal damage. They found that exposure to a single dose of 5 Gy resulted in an increase in MDA level, a decrease in GSH content, and a decrease in CAT activity. Induction of oxidative stress leads to oxidative damage to proteins, lipids, and DNA. In response to DNA damage, DNA damage repair (DDR) pathways are modulated [36]. BRCA1 is a DNA damage response gene that transcriptionally regulates genes involved in DNA damage repair preventing permanent DNA damage. It is also involved in the regulation of cellular redox homeostasis [37]. Our current results revealed a significant decreased in the relative expression of BRCA1 gene in response to gamma radiation exposure and iron overload. These results are consistent with those of Affandi et al. [38], who reported a 70% decrease in BRCA1 gene expression 2 h after ionizing radiation (IR) exposure. DNA damage is a well-established mechanism activated in response to radiation exposure [39]. Several in vitro studies have explained the mechanisms underlying radiation induced DNA damage. Gilman et al. [40] demonstrated that after exposure to IR, the release of ATP could induce the inhibition of DNA repair mechanisms. The other signaling pathway that was described by Hoorelbeke et al. [41] involves P2R signaling molecules, such as nitric oxide (NO). In addition to redox state imbalance, inflammation and angiogenesis are considered well-documented mechanisms associated with radiation induced salivary gland damage [42]. Inflammation and angiogenesis are closely integrated mechanisms [43]. Inflammation is a cellular response in which interleukins and proinflammatory cytokines are secreted from immune cells. TNF-α is considered one of the most representative proinflammatory cytokines and acts through the involvement of ROS generation [44] and activation of NF-κB and IL-1β by binding to the TNF-receptor [45]. Inflammatory cytokines (TNFα and IL-1β) enhance angiogenesis through different molecular pathways, including the upregulation of VEGF [46, 47]. VEGFs are a family of secreted angiogenic growth factors that act specifically on vascular endothelial cells [48]. Our current results confirmed the involvement of these mechanisms in radiation induced salivary gland damage through the significant upregulation of the TNFα, IL-1β, and VEGF genes in the RF group. Our results are in agreement with those of Zhang et al. [49] and I et al. [50] who reported the role of inflammation and angiogenesis in radiation induced salivary gland damage.

In the present study, a potential mechanism for radiation induced salivary gland damage, the PI3K/Akt/mTOR pathway, was demonstrated to be crucial for cell survival, inflammation, and angiogenesis. Ionizing radiation activates PI3K which in turn catalyzes the phosphorylation and translocation of Akt which regulates the mTOR activation through a signal transduction cascade [35]. Our present results revealed significant upregulation of the relative mRNA expression of the PIK3ca, Akt, and mTOR genes in the RF group. Similarly, several previous studies reported the same results in the intestine of rats [35], cultured endothelial cells [51], and in mammary carcinoma cell lines [52]. The PI3K/Akt/mTOR signaling pathway is a vital inflammatory regulator that is involved in the release of cytokines from macrophages [53, 54]. In addition to affecting the expression and secretion of VEGF, it is also involved in the phosphorylation and regulation of BRCA1 protein [55, 56].

Recently flavonoids have attracted widespread-attention due to their radioprotective effects. The radioprotective effect of these compounds could be attributed to their potent antioxidant and anti-inflammatory properties [57]. Flavonoids exert their antioxidant effects by scavenging ROS, and increasing the production of antioxidant enzymes [58]. The radioprotective effects of flavonoids could be attributed to the presence of phenolic hydroxyl groups in their structures [59]. In addition, their effect could also be associated with their ability to downregulate PI3K/Akt/mTOR signaling, as reported by Rahmani et al. [60] and Dong et al. [61]. In the present study, cotreatment of salivary gland with hesperidin and rutin ameliorated the depression of antioxidant mechanisms, DNA damage, inflammation, vasculogenic responses, and PI3K/AKT/mTOR signaling induced by radiation and iron overload.

Hesperidin is a major flavonoid that has a potent radioprotective effect that is mediated by its anti-inflammatory and antioxidant properties. Similarly, Sakat and his colleagues [62] recorded the radioprotective effect of hesperidin against oxidative and inflammatory damage in the submandibular gland. Additionally, the radioprotective effects of hesperidin were reported in mouse testes [63], rat lung tissue [15, 64], rat hepatic tissue [65], bone marrow cells [66], and peripheral blood cells [67].

Another radioprotective flavonoid evaluated in the present study is rutin. Rutin is a potent antioxidant and anti-inflammatory agent. The radioprotective effect of rutin on the salivary gland has not yet been well studied. Although, its radioprotective effect was reported in other in vitro studies by Sunada et al. [68] and Ojha et al. [69], it was also studied in other in vivo studies conducted by Patil et al. [70] and Patil et al. [20].

Our histopathological examination of submandibular glands from the radiation and iron overload groups revealed loss of gland architecture with acinar vacuolization and degeneration, interacinar spaces, hemorrhage and nuclear pyknosis. The ducts showed a loss of normal cellular arrangement with complete degeneration of some of them and some pyknotic, flattened and polymorphic nuclei. Moreover, compared with those of the control, the glands of these rats exhibited significant overexpression of both VEGF and NF-κB. A similar result was reported by Ahmed et al. [71] who reported progressive acinar cytoplasmic vacuoles with pyknotic nuclei in the serous and mucous acini of irradiated submandibular glands. Some ducts appeared degenerated with sloughing of their epithelial lining while others showed stagnation of eosinophilic materials in their lumina. Additionally, gamma irradiated parotid glands exhibited increased interacinar and interlobular spaces, loss of acinar acini architecture, hyperchromatic and pleomorphic acinar nuclei and dilated ducts with stagnant secretion. Compared with those of the control, the glands of these rats showed a significant increase of VEGF [72]. Gamma irradiation induces a significant increase in NF-κB expression in oral mucosal tissue [73] and thekidney [74].

Our results demonstrated that both hesperidin and rutin administration improved the histological structure of submandibular glands subjected to gamma radiation and iron overload. The acinar cell vacuolization, nuclear pyknosis, interacinar spaces, hemorrhage were decreased to a great extent. Most ducts and GCTs restored the normal cellular arrangement. In addition, the submandibular gland VEGF and NF-κB expressions levels were significantly lower as compared to the RF group. The radioprotective effect of hesperidin was demonstrated in the lung [12] as hesperidin administration significantly reduced pulmonary inflammation, edema and fibrosis, alveolar thickness and inflammatory cells induced by radiation. Additionally, hesperidin administration protected irradiated heart tissues by significantly reducing myocardial inflammation, fibrosis and mast cells and macrophages [75]. Moreover, hesperidin reduced NF- κB expression in the testis intoxicated by bisphenol A [76] and in the pancreas intoxicated with cadmium [77]. The effect of hesperidin on iron overload-related oxidative stress was studied by Aalikhani et al. [13], who reported that hesperidin could increase SOD and catalase (CAT) activities in both serum and brain tissue and decrease iron deposition in brain tissue. Additionally, Pari et al. [78] reported that hesperidin administration modulates the increase in the levels of serum hepatic and renal function markers induced by iron overload. Furthermore, hesperidin restored the elevated iron levels to within normal levels. Our results were in accordance with those who reported that rutin significantly reduced NF-κB expression induced by lipopolysaccharide [18] and mercuric chloride [79]. The effect of rutin as a radioprotective agent was evident as reported by El-Ghazaly et al. [80] where rutin administration significantly decreased serum hepatic enzymes, lactate dehydrogenase, TNF-α, nitric oxide, malondialdehyde (MDA), and gastric mucosa myeloperoxidase activity. Aziza et al. [17] demonstrated the effect of rutin against iron overload where rutin administration reduced the serum iron concentration, alanine aminotransferase (ALT) level, aspartate aminotransferase (AST) level, NO level, liver iron concentration and MDA content while the liver glutathione (GSH) level and CAT and SOD activities increased.

There is a great deal of similarity in the mechanisms by which radiation and iron overload damage different organs. Both of these factors enhance free radical generation by inhibiting the activity of free radical scavenging enzymes including SOD, glutathione peroxidase and glutathione S-transferase which are associated with elevated levels of the oxidative stress markers such as MDA and NO [81, 82]. These similarities indicate that both hesperidin and rutin to improve oxidative stress induced by gamma radiation and iron overload. The positive effect of hesperidin in our study could be attributed to its antioxidant activity through direct free radical scavenging, downregulation of prooxidative enzymes that contribute to free radical generation, enhancement of antioxidant enzyme activity and chelation of transition metals [10]. Similarly, rutin improved the oxidative stress induced by gamma radiation and iron overload. This improvement could be attributed to its strong radical scavenging activity, inhibition of lipid peroxidation, effective inhibition of ROS generation and enhancement of the levels and activities of various antioxidants [83, 84].

## Conclusions

We concluded that, hesperidin and rutin can be used as radioprotective and iron chelating agents in irradiated salivary glands affected by iron overload due to their antioxidant, anti-inflammatory, DNA repair, and angiogenesis properties. The limitations of the current study include the fact that the doses of these flavonoids used were studied in rats and the exposure level to these flavonoids in humans must be investigated. Moreover, hesperidin and rutin must be tested at different doses. Thus, we encourage the researchers to continue at these points to fill this gap.

## Data Availability

All data generated or analyzed during this study are included in this published article.
